# Evaluation of quantitative assays for the identification of direct signal transducer and activator of transcription 3 (STAT3) inhibitors

**DOI:** 10.18632/oncotarget.12868

**Published:** 2016-10-25

**Authors:** Steffanie L. Furtek, Christopher J. Matheson, Donald S. Backos, Philip Reigan

**Affiliations:** ^1^ Department of Pharmaceutical Sciences, Skaggs School of Pharmacy and Pharmaceutical Sciences, University of Colorado Anschutz Medical Campus, Aurora, CO, 80045, USA

**Keywords:** STAT3, FP assay, ELISA, lnhibitors, niclosamide

## Abstract

In many forms of cancer the signal transducer and activator of transcription 3 (STAT3) transcription factor remains constitutively active, driving cancer survival and progression. The critical role of STAT3 in tumorigenesis has prompted a campaign of drug discovery programs to identify small molecules that disrupt the function of STAT3, with more recent efforts focusing on direct STAT3 inhibition. There are two target binding sites for direct STAT3 inhibitors: the SH2 dimerization domain and the DNA-binding domain. An *in vitro* fluorescence polarization assay, using recombinant STAT3 protein, has successfully identified compounds that target the SH2 domain; however, no assay has been reported to identify inhibitors that bind the DNA-binding domain. The lack of such a quantitative assay has limited the identification and development of STAT3 DNA-binding domain inhibitors. Here, we report a modified DNA-binding ELISA to incorporate recombinant STAT3 protein to evaluate small molecules that prevent STAT3-DNA binding. The concomitant use of the ELISA and fluorescence polarization assay enables the classification of direct STAT3 inhibitors by their site of action. Our data provide further support that niclosamide inhibits STAT3 through interaction with the DNA-binding domain. Furthermore, the ELISA can support medicinal chemistry efforts by identifying DNA-binding domain inhibitors and allowing the determination of an IC_50_ value, supporting the ranking of inhibitors and development of structure-activity relationships. Therefore, we propose a tandem evaluation approach to identify small molecules that target the SH2 domain or the DNA-binding domain of STAT3, which allows for quantitative evaluation of candidate STAT3 inhibitors.

## INTRODUCTION

Signal transducer and activator of transcription 3 (STAT3) is a member of the STAT family of transcription factors that regulate many cellular processes including cell growth and survival, and immune response [1–3]. The activation of STAT3 is mediated through the binding of cytokines and growth factors that stimulate a signaling cascade resulting in the phosphorylation of Tyr705 within the Src homology 2 (SH2) domain of STAT3, and the formation of active homodimers [4–8]. Active STAT3 dimers translocate from the cytoplasm to the nucleus where they bind DNA and induce transcription of genes involved in the regulation of cell growth and survival [4, 9, 10]. In normal cells this process is transient; however, in many solid and hematological cancers STAT3 remains constitutively active, promoting tumor survival and progression [5, 11]. This critical role in carcinogenesis makes STAT3 as an attractive therapeutic target for treating human malignancies [9, 10, 12, 13].

**Figure 1:**
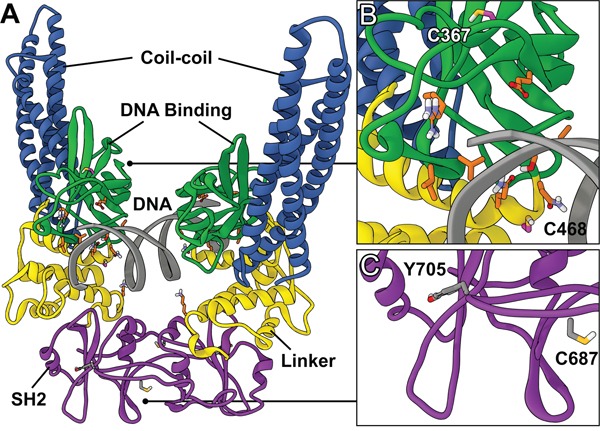
Structure of STAT3 dimer complexed with DNA **A.** Global view of the STAT3 homodimer crystal structure containing DNA (PDB ID: 4E68). **B.** DNA-binding domain. Location of the redox-sensitive C367 and C468 residues (magenta) and residues involved in direct DNA interaction (orange). **C.** SH2 dimerization domain. Location of the redox-sensitive C687 residue and the target of activating phosphorylation Y705.

**Figure 2:**
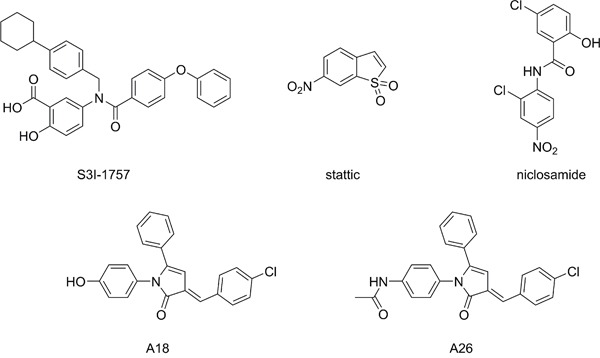
Structures of small molecule STAT3 inhibitors

A number of strategies have been examined to inhibit STAT3 activation, such as targeting the activating JAK2 kinase; however, these broad-spectrum approaches often result in off-target effects [10, 12, 14, 15]. Therefore, there has been recent interest in direct inhibition of STAT3, and this has mainly focused on targeting the SH2 dimerization domain [14, 16]. The SH2 domain is the location of the activating Tyr705 residue and is the protein-protein interface responsible for the formation of transcriptionally active STAT3 dimers (Figure 1). Small molecules designed to target this domain, such as S3I-1757 (Figure 2), aim to disrupt the protein-protein interaction of the SH2 domains of activated STAT3 monomers [12]. Due to the difficulty in developing small molecules capable of disrupting protein-protein interactions over a large surface area, while maintaining drug-like properties, there are a limited number of SH2 domain inhibitors that have reached pre-clinical and clinical trials [10, 15, 17, 18]. Stattic was one of the first small molecule inhibitors of STAT3 to be identified [10, 19], and it was initially believed to be a SH2 domain inhibitor; however, it was later determined that its inhibitory activity was due to modification of the protein through covalent interactions with cysteine residues, many of which reside in the DNA-binding domain (DBD) of STAT3 (Figures 1B and 1C) [20, 21]. Therefore, there has been interest in developing small molecules that disrupt STAT3 transcriptional activity by targeting the DBD of STAT3 (Figure 1) [22–24]. To date, few small molecules have been reported as STAT3 DBD inhibitors, due to the lack of an efficient *in vitro* screening assay, hindering the drug discovery process. It has been proposed that niclosamide, an anthelmintic drug, inhibits the ability of STAT3 to bind DNA [24], and the pyrrolone based compounds A18 and A26 have been reported as STAT3 DBD inhibitors [23] (Figure 2). Targeting this domain may prove more successful in abrogating STAT3 activity in cancer, as these compounds have the potential to inhibit STAT3 transcriptional activity regardless of dimerization status [10]. This prospective success may be due in part to the ability of unphosphorylated STAT3 to be transcriptionally active [18, 25, 26], and the observation that inhibition of active STAT3 dimers alone may not be sufficient in modulating STAT3 activity [23].

The cell-based assays used to evaluate STAT3 inhibition with small molecules include the STAT3-dependent dual luciferase reporter assay [27, 28], electromobility shift assay (EMSA) [22, 24, 26], and measuring expression of gene targets downstream of STAT3 signaling [23, 29]. These assays effectively evaluate the inhibitory potential of small molecules against STAT3 signaling; however, they provide little to no information regarding their site of action or if the inhibition is a direct result of STAT3 inhibition [10]. For the purposes of STAT3 inhibitor design and development, these cell-based methods of evaluation are uninformative without *in vitro* assays with the capacity to determine direct inhibition of STAT3-DNA binding, the potency of inhibition, and the site of interaction. The *in vitro* fluorescence polarization (FP) assay, developed by Schust and Burg [30], allows for rapid identification of small molecule inhibitors of recombinant STAT3 acting at the SH2 domain. Although the FP assay is capable of identifying STAT3 inhibitors that act at the SH2 domain, it does not identify small molecules that target the DBD of STAT3. Nkansah *et al*., have demonstrated that unphosphorylated STAT3 (uSTAT3) is capable of binding its corresponding DNA sequence using a protein electrophoretic mobility shift assay (PEMSA), supporting the use of recombinant STAT3 for the evaluation of DBD inhibition; however, small molecule inhibitors were not evaluated *via* this method [26]. In this manuscript we introduce a modified *in vitro* DNA-binding STAT3 ELISA as part of a tandem evaluation approach to elucidate and quantitatively assess small molecule inhibitors that target either the SH2 dimerization domain or the DBD of STAT3.

## RESULTS

### The STAT3 inhibitors S3I-1757 and A26 bind the SH2 domain of STAT3 in an FP assay

**Figure 3:**
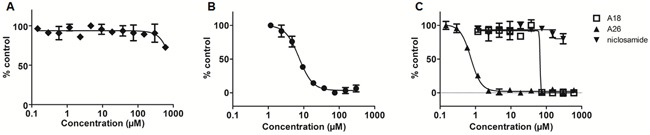
Fluorescence polarization (FP) assay measures affinity of STAT3 inhibitors for the SH2 domain **A.** stattic **B.** S3I-1757, and **C.** A18, A26, and niclosamide.

The FP assay assesses the ability of a compound to bind the SH2 domain of recombinant STAT3 and disrupt fluorescein-labeled peptide binding [30]. Stattic inhibits STAT3 activity *via* cysteine alkylation [21] and despite a cysteine residue residing in the SH2 domain (Cys687), we did not detect any disruption of STAT3-peptide binding with stattic up to 600μM (Figure 3A). S3I-1757 has been reported as an SH2 domain inhibitor of STAT3 and our results support that S3I-1757 can bind the SH2 domain and prevent protein-protein interactions at this interface, with an IC_50_ of 7.39 ± 0.95μM in the FP assay (Figure 3B). Both compounds A18 and A26 have been reported as DBD inhibitors of STAT3 [23]. A18 demonstrated no activity in the FP assay; however, A26 exhibited robust inhibition with an IC_50_ value of 0.74 ± 0.13μM (Figure 3C). Niclosamide has also been proposed to target STAT3; however, its mode of action has not been confirmed although suggested to target the DBD. From our results, niclosamide does not interact with the SH2 domain at concentrations up to 600μM (Figure 3C). The compounds that demonstrate no activity in the FP assay (up to 50μM) require an alternative *in vitro* assay to determine if they exhibit inhibitory action on the DBD of STAT3.

### Recombinant, full-length STAT3 demonstrates a concentration-dependent increase in DNA-binding in a modified ELISA

**Figure 4:**
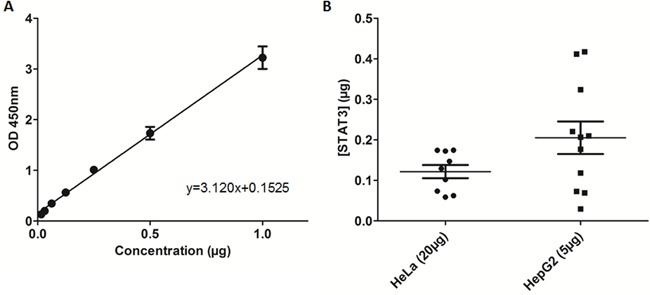
Recombinant STAT3 ELISA evaluation and quantification of nuclear STAT3 bound to DNA in cell extracts **A.** Recombinant STAT3 added to TransAM STAT3 ELISA in increasing concentrations and plotted based on optical density (OD450). **B.** Quantification of nuclear extract samples from loading either 5μg of HepG2 nuclear extracts or 20μg of HeLa nuclear extracts.

The STAT3 DNA-binding ELISA can be used to evaluate the ability of STAT3 in cellular nuclear extracts to bind its corresponding consensus sequence that has been immobilized on a 96-well plate [31, 32]. Addition of full-length, recombinant monomeric STAT3 to the TransAM STAT3 ELISA resulted in a concentration-dependent increase in optical density (OD). These results demonstrate that OD (y) exhibited a linear correlation with the concentration of STAT3 (x), given by the equation *y* = 3.120*x*+0.1525 (Figure 4A). The *in vitro* ELISA results demonstrated that recombinant STAT3 had measurable binding activity and suggested that incorporating recombinant STAT3 into the assay may allow for more accurate and direct assessment of STAT3 inhibitors in a cell-free system.

### Quantification of STAT3-DNA binding in nuclear extracts

Using the equation obtained in the previous section, we quantified STAT3 in nuclear extract samples that bound the DNA consensus sequence immobilized on the ELISA plate. The TransAM STAT3 ELISA kit provides prepared nuclear extracts of HepG2 cells stimulated with IL-6 (100 ng/mL). The average OD recorded for the recommended 5μg total protein of HepG2 nuclear extracts was 0.793, equating to an average STAT3-DNA bound content of 0.21μg (Figure 4B), although there was substantial variability among the different samples. The average OD recorded for 20μg total protein of HeLa nuclear extracts was 0.532, equating to an average STAT3-DNA bound content of 0.12μg (Figure 4B). Based on data consistency, HeLa cells were used for the evaluation of inhibitors in cell-based assays. Furthermore, the data obtained from the nuclear extracts served as a protein loading guide based on OD.

### Stattic, S3I-1757, and niclosamide inhibit recombinant STAT3-DNA binding in a DNA-binding ELISA

**Figure 5:**
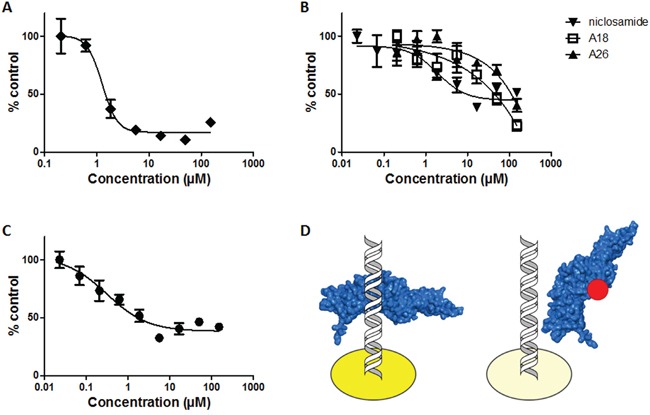
Recombinant STAT3 ELISA reveals STAT3 inhibitors that act at the DNA-binding domain Recombinant STAT3 incubated for 1h with **A.** stattic, **B.** A18, A26, niclosamide, or **C.** S3I-1757 prior to addition to ELISA plate, and **D.** schematic of modified TransAM STAT3 ELISA using recombinant STAT3. *Left*: Control well demonstrating binding of recombinant STAT3 (blue) to corresponding DNA consensus sequence to produce maximum OD450 (dark yellow). *Right*: Representative experimental well demonstrating interruption of STAT3 (blue) binding to DNA in the presence of a DBD inhibitor (red) resulting in reduced OD450 (light yellow).

Recombinant STAT3 was incorporated into a STAT3-DNA binding ELISA to determine small molecule binding and disruption of STAT3-DNA binding (Figure 5D). The cysteine alkylator stattic inhibited STAT3-DNA binding with an IC_50_ of 1.27 ± 0.38μM (Figure 5A). S3I-1757, which also demonstrated affinity for the SH2 domain, inhibited STAT3-DNA binding with an IC_50_ value of 0.31 ± 0.18μM (Figure 5C). A26 and A18 demonstrated weak inhibition of STAT3-DNA binding at concentrations greater than 10μM and no IC_50_ values could be determined (Figure 5B). Niclosamide exhibited inhibition of STAT3-DNA binding with an IC_50_ of 1.93 ± 0.70μM, supporting its site of action as the DBD of STAT3 (Figure 5B). The compounds that demonstrate activity in the FP assay but not the ELISA prevent STAT3 dimerization, those that have activity in the ELISA assay but not the FP assay prevent direct interaction of STAT3 with DNA, and compounds that have activity in both the ELISA and the FP assay are mixed mode inhibitors. Once the mode of STAT3 inhibition is known compounds can then be evaluated in cell-based assays, even compounds that demonstrate no activity in the FP or ELISA assays could be tested in the cell-based assays to determine indirect effects on STAT3 activity.

### Niclosamide demonstrates potent inhibition of STAT3 in cell-based ELISA at sublethal concentrations

**Figure 6:**
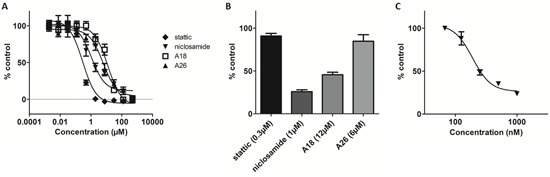
Cell-based STAT3 ELISA demonstrates inhibition of STAT3 with niclosamide at sub-lethal concentrations **A.** MTT of HeLa cells incubated with compounds for 24h. **B.** Cell-based ELISA of HeLa cells dosed for 24h at EC_50_ as determined by MTT. **C.** HeLa cells dosed with niclosamide (0.0675μM-1.0μM) for 24h and STAT3 DNA-binding determined by ELISA.

The effect of the various STAT3 inhibitors on cell viability after 24-hour treatment was assessed by MTT assay and the EC_50_ of each compound was determined: stattic 0.29 ± 0.09μM, A18 12.39 ± 1.2μM, A26 6.10 ± 1.3μM, and niclosamide 1.09 ± 0.9μM (Figure 6A). Similar to an EMSA, the STAT3 DNA-binding ELISA measures the ability of activated STAT3 present in nuclear extracts to bind to its corresponding DNA consensus sequence [31–34]. Therefore, an ELISA performed on cellular nuclear extracts allows for the evaluation of compounds that target the SH2 domain and/or the DBD of STAT3. STAT3 inhibitory potential of the compounds was assessed by incubating HeLa cells at concentrations capable of decreasing cell viability by 50% as determined by MTT, followed by preparation of nuclear extracts to evaluate by ELISA. Stattic and A26 did not alter STAT3-DNA binding at their respective EC_50_ concentrations (Figure 6B). In contrast, both A18 and niclosamide reduced STAT3-DNA binding compared to control (55% and 75%, respectively) (Figure 6B). An EC_50_ of 0.19 ± 0.001μM was determined from a dose-response curve for niclosamide in analysis of nuclear extracts in the ELISA (Figure 6C).

## DISCUSSION

Direct small molecule inhibitors of STAT3 activity have diverse chemical structures and vary by mode of action. Inhibitors of STAT3, particularly those that target STAT3-DNA binding, have been evaluated using cell-based assays, but the results of these assays can be difficult to interpret as the mode of action of the compound and the manner in which it directly or indirectly affects STAT3 cannot be determined *via* these methods. Furthermore, IC_50_ values for compounds tested in cell-based assays vary between assay and cell-type and may not be representative of the effects of STAT3 inhibition alone [10]. Therefore, the expansion of *in vitro* assays available to evaluate direct STAT3 inhibition and inhibitor mode of action is critical for medicinal chemistry efforts focused on the advancement of STAT3 inhibitor identification and development.

A well-established method for evaluating small molecule inhibitors of the SH2 domain is the FP assay [30]. This competitive binding assay uses monomeric recombinant STAT3 and a fluorescent peptide that has high affinity for the STAT3 SH2 domain [30]. Small molecules targeting this domain impair the binding of the peptide, leading to a change in fluorescence based on free versus bound peptide. In the FP assay, stattic did not present any observable activity against the SH2 domain up to 600μM. Cys687 within the SH2 domain and both Cys367 and Cys468 within the DBD, are all potential targets for stattic and alkylation within the DBD may be the primary mechanism of action of stattic-mediated STAT3 inhibition [21, 35]. The lack of inhibition of stattic at the SH2 domain may be due in part to the inability of stattic to disrupt the protein-peptide interaction through the alkylation of Cys687 alone. Similarly, the steric bulk of stattic may be insufficient to disrupt STAT3 dimerization. S3I-1757 has been reported as a STAT3 SH2 domain inhibitor and has previously been evaluated in a FP assay [17]. The IC_50_ of 7.39 ± 0.95μM value obtained from our FP assay data, is in good agreement with values reported in the literature [17], and S3I-1757 served as a positive control for this assay. Both A18 and A26 have been reported as DBD inhibitors [23], but the activity of A18 or A26 has not been previously reported in the FP assay and, as expected, A18 exhibited no inhibition in the FP assay. Interestingly, despite significant structural similarities with A18, A26 demonstrated good affinity for the SH2 domain with an IC_50_ value of 0.74 ± 0.13μM. Previous studies utilized immobilized A26 in a protein pull-down experiment with various domain deletions of recombinant STAT3 protein to determine the DBD was the binding site of A26. The results of our FP assay suggest that this compound may have a bimodal mechanism of action with the capacity to bind both the SH2 and DBD sites of STAT3. In contrast, the mechanism of STAT3 inhibition by niclosamide had previously been proposed to be due to targeting the DBD based on results from EMSA analysis [24]. Our results further support that niclosamide does not have substantive affinity for the SH2 domain and exerts its inhibitory effects *via* interaction with the DBD, preventing DNA binding and subsequent transcriptional activation.

It has previously been confirmed that recombinant STAT3 can bind to DNA and the interaction of uSTAT3 with DNA is similar to the binding orientation of active pSTAT3 [26]. The recombinant STAT3 ELISA results demonstrated the suitability of this assay to both determine the binding of recombinant STAT3 protein and identify potential inhibitors of STAT3-DNA binding. The capability of full-length recombinant STAT3 to bind its corresponding consensus DNA sequence immobilized on an ELISA plate allows the estimation of the amount of activated STAT3 bound to DNA in nuclear extract samples. Using the concentration of STAT3 quantified in HeLa nuclear extracts as a guide, compounds were evaluated for their DNA-binding inhibitory activity against monomeric recombinant STAT3 in the modified ELISA. The compounds selected in our study inhibit STAT3 activity with differing modes of action and display inhibition of STAT3 in STAT3-dependent luciferase reporter assays [17, 23, 24, 36]. Although assay conditions are not uniform across publications, all compounds examined in our study reduced STAT3 transcriptional activity to varying degrees.

Stattic alkylates cysteine residues in the DBD of STAT3 [21, 35], resulting in the decreased activity observed in the recombinant protein ELISA. S3I-1757 also showed potent activity in the ELISA against STAT3 monomers and this may be due to S3I-1757 having a bimodal mechanism of action, acting at both the SH2 domain and DBD. Niclosamide, A18, and A26 have been suggested as DBD inhibitors, yet only niclosamide demonstrated appreciable activity in the recombinant protein ELISA assay. Our results indicate that A18 and A26 do not effectively inhibit STAT3 *via* binding to the DBD. A26 exhibited potent activity in the FP assay and little activity in the *in vitro* ELISA at concentrations up to 50μM. In contrast to previous reports, A18 had no observable activity in either the FP or the *in vitro* ELISA assays up to 10μM, suggesting the mechanism of action of A18 is not a result of direct STAT3 inhibition, despite structural similarity to A26. Since the *in vitro* ELISA using monomeric recombinant STAT3 does not rely on the formation of STAT3 dimers, the primary use of this ELISA method is to identify potent inhibitors that target the DBD as opposed to the SH2 domain of STAT3, when used in combination with the FP assay. Structural analysis studies using X-ray crystallography may further confirm the interaction of niclosamide at the DBD; however, there are only 3 crystal structures (PDB ID: 4E68 [26], 3CWG [37], and 1BG1 [38]) of the core STAT3 fragment containing the SH2 domain and DBD currently deposited in the Protein Data Bank (http://www.rcsb.org) and no STAT3-inhibitor co-crystal structures are present, suggesting that the resolution of the STAT3 crystal structure is not a trivial endeavor.

**Figure 7:**
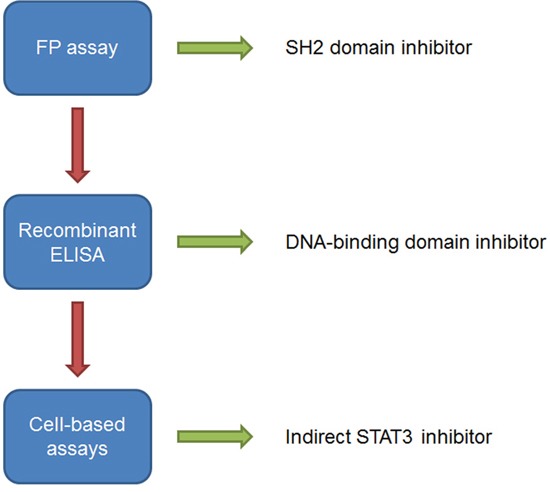
Workflow for the evaluation of STAT3 small molecule inhibitors to determine site of action Arrows indicate assay result as either activity as an inhibitor of STAT3 (green) or no activity (red) in the respective assay. Compounds that demonstrate activity in the FP assay and ELISA using recombinant STAT3 protein should also be evaluated in cell systems, such as the ELISA that detects STAT3 activity in nuclear extracts, to determine the effect of STAT3 inhibition in cells.

The MTT assay was used to determine the maximum concentration of compound that could be used in cells prior to the preparation of cellular nuclear extracts without depleting cell numbers such that isolated protein concentrations reside outside of those required for detection in the ELISA assay. Dosing cells at a concentration greater than the EC_50_ would result in too few cells remaining to produce the required 20μg of total protein in the nuclear extracts. Although stattic demonstrated inhibition of STAT3-DNA binding in the ELISA using recombinant STAT3, no inhibition was observed in the ELISA using nuclear extracts, possibly due to the non-specific cysteine alkylation of other cellular proteins. S3I-1757 has previously displayed high micromolar activity in cells [17], and no EC_50_ could be determined from the MTT assay (data not shown). Therefore, S3I-1757 was not advanced forward for the evaluation of STAT3 inhibition by ELISA in HeLa cells. A18 induced a ~55% reduction in STAT3-DNA binding at its MTT derived EC_50_ of 12μM while A26 displayed no significant activity at the corresponding concentration of 6μM. Results reported for a STAT3 EMSA in H1299 cells is consistent with a 50% reduction of STAT3-DNA binding for A18 at 12μM and no reduction in binding for A26 at 6μM [23]. The lack of activity of A18 in the FP assay and the *in vitro* ELISA using recombinant STAT3, but activity in the cell-based ELISA supports a mode of action other than direct STAT3 inhibition (Figure 7). This highlights the need for cell-free systems to evaluate direct STAT3 inhibitors, as cell-based assay can provide confounding results, where compounds may have direct or indirect effect on STAT3. Niclosamide demonstrated potent inhibition of STAT3 in the cell-based ELISA with an EC_50_ of 0.19 ± 0.001μM. The increased potency of niclosamide in the cell-based ELISA suggests that niclosamide may also target other members upstream of STAT3, resulting in a greater impact on STAT3-DNA binding. This is further supported in the literature, as niclosamide has been investigated for its anticancer activity in additional signaling cascades many of which crosstalk with the STAT3 signaling pathway [39–42].

Taken together, our data support that the FP assay and ELISA using recombinant STAT3 can serve as an effective quantitative approach to identify potent direct inhibitors of STAT3 acting at the SH2 dimerization domain or the DBD (Figure 7). The use of the FP assay and the recombinant STAT3 ELISA has a number of advantages over cell-based methods: 1) it allows the identification of direct STAT3 inhibitors, 2) by using both assays the site of interaction can be determined, and 3) the assays provides dose-response and IC_50_ data for STAT3 inhibitors allowing an evaluation of inhibitory potency; therefore, compounds can be ranked and structure-activity relationships (SARs) can be established. Furthermore, the data obtained from the ELISA using nuclear extracts from HeLa cells provides initial support that STAT3 inhibitors targeting the DBD are more efficient than those targeting the SH2 domain in cellular systems. In this study we have established an approach for identifying DBD inhibitors of STAT3 and our data strongly supports that niclosamide targets the DBD of STAT3. Although niclosamide was not designed or developed as a STAT3 inhibitor, it may provide a structural basis for the development of more selective STAT3 inhibitors targeting the DBD.

## MATERIALS AND METHODS

### Cells and reagents

Human cervical carcinoma cells (HeLa) were obtained from ATCC and were verified by the vendor. Cells were grown in Eagle's Minimum Essential Medium (EMEM) supplemented with 10% heat inactivated FBS. Compounds S3I-1757 (AOBIOUS), stattic (ApexBio Tech LLC), and niclosamide (Cayman Chemical Co.) were purchased. Compounds A18 and A26 were synthesized according to previously reported methods [23, 43].

### FP assay

Fluorescence polarization (FP) assay was conducted as previously reported [17, 30]. Briefly, assay buffer (10 mM HEPES, pH7.5, 50 mM NaCl, 1 mM EDTA, 2 mM DTT, and 0.01% Triton-X100), 100 nM of full length GST-tagged human STAT3 protein (abcam), and varying concentrations of S3I-1757, A18, A26, stattic, or niclosamide were incubated for 1 hour at room temperature with mild agitation in 96-well half area black plates (Corning). Fluorescent peptide 5-FAM-G(pTyr) LPQTV-CONH_2_ (Genscript) was added to each assay well at a concentration of 10 nM. Final well volumes were 30μL. Following incubation with fluorescent peptide for 30 minutes at room temperature with mild agitation, plates were examined using FP Fluorescein Dual module with excitation filter FITC FP 480 and emission filter FITC FP P-pol535 and S-pol535. Data was expressed as percent control using the equation %C = (mP_drug_-mP_free_)/(mP_STAT3_-mP_free_)*100 where mP is the value for FP measurement.

### Recombinant STAT3 ELISA

Measurement of STAT3 binding to DNA was performed using a TransAM STAT3 ELISA kit (Active Motif). The TransAM STAT3 ELISA kit provides oligonucleotides containing the STAT3 consensus binding sequence immobilized on the ELISA plates. The kit was utilized according to product directions with the replacement of cellular nuclear extracts with recombinant STAT3 protein. Full-length, GST-tagged recombinant human STAT3 protein (abcam) was incorporated into the TransAM STAT3 ELISA kit in increasing amounts (0.01μg–1.0μg). To each well to be used, 30μL of Complete Binding Buffer was added. To the sample wells was added recombinant STAT3 diluted in 20μL of Complete Lysis Buffer and the plate was incubated at room temperature with mild agitation for 1 hour. A blank well containing 20μL of Complete Lysis Buffer was also included. Following the 1 hour incubation, wells were washed 3 times with 200μL of 1X Wash Buffer and then incubated with STAT3 antibody (1:1000 dilution) in 100uL of 1X Antibody Binding Buffer for 1 hour without agitation. Wells were washed 3 times with 200μL of 1X Wash Buffer and incubated with HRP-conjugated antibody (1:1000 dilution) in 100uL of 1X Antibody Binding Buffer for 1 hour without agitation. Finally, wells were washed 4 times with 200μL of 1X Wash Buffer and then developed using room-temperature Developing Solution for 15 minutes. Stop Solution was added and the plate was immediately read for absorbance at 450 nm with reference wavelength at 655 nm on a microplate reader.

### Compound evaluation

Full-length, GST-tagged human STAT3 protein and various concentrations of S3I-1757, A18, A26, stattic, or niclosamide were incubated in Complete Lysis Buffer at a final volume of 20μL containing 0.08μg recombinant protein for 1 hour with mild agitation. Following incubation with STAT3 inhibitors, samples were added to corresponding ELISA wells with 30μL of Complete Binding Buffer and inhibition of STAT3 protein binding to the DNA consensus sequence was determined *via* the previously mentioned method. Data was normalized and reported as percent of control.

### MTT assay

HeLa cells were plated in 96-well plates at a density of 5,000 cells/well in 100μL of media and were incubated for 24 hours. Media was then aspirated and replaced with 100μL of media containing various concentrations of compounds ranging from 500μM-0.0019μM. Cells were exposed to compounds for 24 hours, following which the media was aspirated and 50μL MTT solution diluted in media was added to each well at a final concentration of 1 mg/mL. MTT was exposed to cells for 4 hours, aspirated, and then 100μL of DMSO was added to each well. Plates were agitated for 10 minutes prior to reading absorbance at 540 nm in a microplate reader. Data was normalized and reported as percent of control.

### Nuclear extract preparation

Nuclear extracts were prepared using a nuclear extraction kit (Signosis) according to product directions. Briefly, 100 mm dishes of cells were washed with 1X PBS and then 1 mL of 1X Buffer I was added and dishes were rocked on ice for 10 minutes. Cells were released from the dish using a sterile scraper and centrifuged at 1200rpm for 5 minutes at 4°C. The supernatant was discarded and the pellets were resuspended in 1X Buffer II and shaken on ice for 2 hours. Samples were centrifuged at 1200rpm for 5 minutes at 4°C and the resulting supernatants containing the nuclear extracts were placed in new microcentrifuge tubes. Protein concentrations of samples were determined *via* Bradford assay.

### Cell-based STAT3 DNA-binding ELISA

HeLa cells were plated at 1 million cells per 100 mm dish and incubated for approximately 72 hours, resulting in serum-starved confluent plates. Plates were then washed with PBS and fresh media was added containing concentrations of A18, A26, stattic, or niclosamide for 24 hours. Cells were treated with compound concentrations derived from their 24 hour MTT assay EC_50_ value. The dose-response of niclosamide was similarly prepared at concentrations ranging from 1.0μM-0.0675μM. Following incubation with compounds, cellular nuclear extracts were prepared.

Measurement of STAT3 binding to DNA was performed using the TransAM STAT3 ELISA kit. Sample wells contained 20μg of nuclear extracts from treated HeLa cells diluted in 20μL of Complete Lysis Buffer. The ELISA was then performed as described in the recombinant ELISA section. Data was normalized and reported as percent of control.

### Statistical analysis

Statistical analysis was performed using GraphPad Prism 5.0, data for IC_50_ and EC_50_ curves was normalized, and error bars represent the standard error of the mean (SEM). Experiments were repeated in triplicate.

## References

[1] Darnell JE (1997). STATs and gene regulation. Science.

[2] Zhang X, Yue P, Fletcher S, Zhao W, Gunning PT, Turkson J (2010). A novel small-molecule disrupts Stat3 SH2 domain-phosphotyrosine interactions and Stat3-dependent tumor processes. Biochemical pharmacology.

[3] Furtek SL, Backos DS, Matheson CJ, Reigan P (2016). Strategies and approaches of targeting STAT3 for cancer treatment. ACS chemical biology.

[4] Schust J, Sperl B, Hollis A, Mayer TU, Berg T (2006). Stattic: a small-molecule inhibitor of STAT3 activation and dimerization. Chemistry & biology.

[5] Lin L, Hutzen B, Li PK, Ball S, Zuo M, DeAngelis S, Foust E, Sobo M, Friedman L, Bhasin D, Cen L, Li C, Lin J (2010). A novel small molecule, LLL12, inhibits STAT3 phosphorylation and activities and exhibits potent growth-suppressive activity in human cancer cells. Neoplasia.

[6] Xiong A, Yang Z, Shen Y, Zhou J, Shen Q (2014). Transcription Factor STAT3 as a Novel Molecular Target for Cancer Prevention. Cancers.

[7] Debnath B, Xu S, Neamati N (2012). Small molecule inhibitors of signal transducer and activator of transcription 3 (Stat3) protein. Journal of medicinal chemistry.

[8] Zhuang S (2013). Regulation of STAT signaling by acetylation. Cellular signalling.

[9] Wang X, Crowe PJ, Goldstein D, Yang JL (2012). STAT3 inhibition, a novel approach to enhancing targeted therapy in human cancers (review). International journal of oncology.

[10] Furtek SL, Backos DS, Matheson CJ, Reigan P (2016). Strategies and Approaches of Targeting STAT3 for Cancer Treatment. ACS chemical biology.

[11] Page BD, Fletcher S, Yue P, Li Z, Zhang X, Sharmeen S, Datti A, Wrana JL, Trudel S, Schimmer AD, Turkson J, Gunning PT (2011). Identification of a non-phosphorylated, cell permeable, small molecule ligand for the Stat3 SH2 domain. Bioorganic & medicinal chemistry letters.

[12] Yue P, Turkson J (2009). Targeting STAT3 in cancer: how successful are we?. Expert opinion on investigational drugs.

[13] Wake MS, Watson CJ (2015). STAT3 the oncogene - still eluding therapy?. The FEBS journal.

[14] Morlacchi P, Robertson FM, Klostergaard J, McMurray JS (2014). Targeting SH2 domains in breast cancer. Future medicinal chemistry.

[15] Miklossy G, Hilliard TS, Turkson J (2013). Therapeutic modulators of STAT signalling for human diseases. Nat Rev Drug Discov.

[16] Fletcher S, Drewry JA, Shahani VM, Page BD, Gunning PT (2009). Molecular disruption of oncogenic signal transducer and activator of transcription 3 (STAT3) protein. Biochemistry and cell biology = Biochimie et biologie cellulaire.

[17] Zhang X, Sun Y, Pireddu R, Yang H, Urlam MK, Lawrence HR, Guida WC, Lawrence NJ, Sebti SM (2013). A novel inhibitor of STAT3 homodimerization selectively suppresses STAT3 activity and malignant transformation. Cancer research.

[18] Botta A, Sirignano E, Popolo A, Saturnino C, Terracciano S, Foglia A, Sinicropi MS, Longo P, Di Micco S (2015). Identification of Lead Compounds as Inhibitors of STAT3: Design, Synthesis and Bioactivity. Molecular informatics.

[19] Kraskouskaya D, Duodu E, Arpin CC, Gunning PT (2013). Progress towards the development of SH2 domain inhibitors. Chemical Society reviews.

[20] McMurray JS (2006). A new small-molecule Stat3 inhibitor. Chemistry & biology.

[21] Heidelberger S, Zinzalla G, Antonow D, Essex S, Basu BP, Palmer J, Husby J, Jackson PJ, Rahman KM, Wilderspin AF, Zloh M, Thurston DE (2013). Investigation of the protein alkylation sites of the STAT3:STAT3 inhibitor Stattic by mass spectrometry. Bioorganic & medicinal chemistry letters.

[22] Huang W, Dong Z, Wang F, Peng H, Liu JY, Zhang JT (2014). A small molecule compound targeting STAT3 DNA-binding domain inhibits cancer cell proliferation, migration, and invasion. ACS chemical biology.

[23] Huang W, Dong Z, Chen Y, Wang F, Wang CJ, Peng H, He Y, Hangoc G, Pollok K, Sandusky G, Fu XY, Broxmeyer HE, Zhang ZY, Liu JY, Zhang JT (2015). Small-molecule inhibitors targeting the DNA-binding domain of STAT3 suppress tumor growth, metastasis and STAT3 target gene expression *in vivo*. Oncogene.

[24] Ren X, Duan L, He Q, Zhang Z, Zhou Y, Wu D, Pan J, Pei D, Ding K (2010). Identification of Niclosamide as a New Small-Molecule Inhibitor of the STAT3 Signaling Pathway. ACS medicinal chemistry letters.

[25] Timofeeva OA, Chasovskikh S, Lonskaya I, Tarasova NI, Khavrutskii L, Tarasov SG, Zhang X, Korostyshevskiy VR, Cheema A, Zhang L, Dakshanamurthy S, Brown ML, Dritschilo A (2012). Mechanisms of unphosphorylated STAT3 transcription factor binding to DNA. The Journal of biological chemistry.

[26] Nkansah E, Shah R, Collie GW, Parkinson GN, Palmer J, Rahman KM, Bui TT, Drake AF, Husby J, Neidle S, Zinzalla G, Thurston DE, Wilderspin AF (2013). Observation of unphosphorylated STAT3 core protein binding to target dsDNA by PEMSA and X-ray crystallography. FEBS letters.

[27] Turkson J, Bowman T, Garcia R, Caldenhoven E, De Groot RP, Jove R (1998). Stat3 activation by Src induces specific gene regulation and is required for cell transformation. Molecular and cellular biology.

[28] Lin L, Hutzen B, Zuo M, Ball S, Deangelis S, Foust E, Pandit B, Ihnat MA, Shenoy SS, Kulp S, Li PK, Li C, Fuchs J, Lin J (2010). Novel STAT3 phosphorylation inhibitors exhibit potent growth-suppressive activity in pancreatic and breast cancer cells. Cancer research.

[29] Hsieh FC, Cheng G, Lin J (2005). Evaluation of potential Stat3-regulated genes in human breast cancer. Biochemical and biophysical research communications.

[30] Schust J, Berg T (2004). A high-throughput fluorescence polarization assay for signal transducer and activator of transcription 3. Analytical biochemistry.

[31] Lafarge S, Hamzeh-Cognasse H, Chavarin P, Genin C, Garraud O, Cognasse F (2007). A flow cytometry technique to study intracellular signals NF-κB and STAT3 in peripheral blood mononuclear cells. BMC Molecular Biology.

[32] Jiang H, Yu J, Guo H, Song H, Chen S (2008). Upregulation of survivin by leptin/STAT3 signaling in MCF-7 cells. Biochemical and biophysical research communications.

[33] Liu LJ, Leung KH, Chan DSH, Wang YT, Ma DL, Leung CH (2014). Identification of a natural product-like STAT3 dimerization inhibitor by structure-based virtual screening. Cell Death Dis.

[34] Mayer C, Gruber HJ, Landl EM, Pailer S, Scharnagl H, Truschnig-Wilders M, Marz W (2007). Rosuvastatin reduces interleukin-6-induced expression of C-reactive protein in human hepatocytes in a STAT3- and C/EBP-dependent fashion. International journal of clinical pharmacology and therapeutics.

[35] Buettner R, Corzano R, Rashid R, Lin J, Senthil M, Hedvat M, Schroeder A, Mao A, Herrmann A, Yim J, Li H, Yuan YC, Yakushijin K (2011). Alkylation of cysteine 468 in Stat3 defines a novel site for therapeutic development. ACS chemical biology.

[36] Spitzner M, Roesler B, Bielfeld C, Emons G, Gaedcke J, Wolff HA, Rave-Frank M, Kramer F, Beissbarth T, Kitz J, Wienands J, Ghadimi BM, Ebner R, Ried T, Grade M (2014). STAT3 inhibition sensitizes colorectal cancer to chemoradiotherapy *in vitro* and *in vivo*. International journal of cancer.

[37] Ren Z, Mao X, Mertens C, Krishnaraj R, Qin J, Mandal PK, Romanowski MJ, McMurray JS, Chen X (2008). Crystal structure of unphosphorylated STAT3 core fragment. Biochemical and biophysical research communications.

[38] Becker S, Groner B, Muller CW (1998). Three-dimensional structure of the Stat3beta homodimer bound to DNA. Nature.

[39] Walters Haygood CL, Arend RC, Gangrade A, Chettiar S, Regan N, Hassmann CJ, Li PK, Hidalgo B, Straughn JM, Buchsbaum DJ (2015). Niclosamide Analogs for Treatment of Ovarian Cancer. International journal of gynecological cancer.

[40] Pan JX, Ding K, Wang CY (2012). Niclosamide, an old antihelminthic agent, demonstrates antitumor activity by blocking multiple signaling pathways of cancer stem cells. Chinese journal of cancer.

[41] Li Y, Li PK, Roberts MJ, Arend RC, Samant RS, Buchsbaum DJ (2014). Multi-targeted therapy of cancer by niclosamide: A new application for an old drug. Cancer letters.

[42] Moskaleva EY, Perevozchikova VG, Zhirnik AS, Severin SE (2015). Molecular mechanisms of niclosamide antitumor activity. Biomeditsinskaia khimiia.

[43] Adam J-mrdV-ND, Rosenau, F-68128, FR), Dalvi, Pramod V. (F3/6 Atic Colony, Dist. Valsad State Gujarat, Atul 0, 396 02, IN), Ekkundi, Vadiraj Subbanna (Herrenweg 60, Aesch, CH-4147, CH), Bacher, Jean-pierre (rue des Vergers 9, Buschwiller, F-68220, FR), Tiwari, Sandip (1020 Aecs Layout, II Main II Cross, D Bloc, Kundanhalli Bangalore 7, 56003, IN). (2004). COMPOUNDS, A PROCESS FOR THEIR PREPARATION AND THEIR USE AS DYES AND PIGMENTS. CIBA SPECIALTY CHEMICALS HOLDING INC. (Klybeckstrasse 141, Basel, CH-4057, CH), Adam, Jean-marie (rue de Village-Neuf 60 D, Rosenau, F-68128, FR), Dalvi, Pramod V. (F3/6 Atic Colony, Dist. Valsad State Gujarat, Atul 0, 396 02, IN), Ekkundi, Vadiraj Subbanna (Herrenweg 60, Aesch, CH-4147, CH), Bacher, Jean-pierre (rue des Vergers 9, Buschwiller, F-68220, FR), Tiwari, Sandip (1020 Aecs Layout, II Main II Cross, D Bloc, Kundanhalli Bangalore 7, 56003, IN))

